# Retrosplenial and postsubicular head direction cells compared during visual landmark discrimination

**DOI:** 10.1177/2398212817721859

**Published:** 2017-09-15

**Authors:** Yave Roberto Lozano, Hector Page, Pierre-Yves Jacob, Eleonora Lomi, James Street, Kate Jeffery

**Affiliations:** Division of Psychology and Language Sciences, University College London, London, UK

**Keywords:** Head direction cells, postsubiculum, retrosplenial cortex, landmarks, visual discrimination, spatial memory, in vivo rodent electrophysiology

## Abstract

**Background:**

Visual landmarks are used by head direction (HD) cells to establish and help update the animal’s representation of head direction, for use in orientation and navigation. Two cortical regions that are connected to primary visual areas, postsubiculum (PoS) and retrosplenial cortex (RSC), possess HD cells: we investigated whether they differ in how they process visual landmarks.

**Methods:**

We compared PoS and RSC HD cell activity from tetrode-implanted rats exploring an arena in which correct HD orientation required discrimination of two opposing landmarks having high, moderate or low discriminability.

**Results:**

RSC HD cells had higher firing rates than PoS HD cells and slightly lower modulation by angular head velocity, and anticipated actual head direction by ~48 ms, indicating that RSC spiking leads PoS spiking. Otherwise, we saw no differences in landmark processing, in that HD cells in both regions showed equal responsiveness to and discrimination of the cues, with cells in both regions having unipolar directional tuning curves and showing better discrimination of the highly discriminable cues. There was a small spatial component to the signal in some cells, consistent with their role in interacting with the place cell navigation system, and there was also slight modulation by running speed. Neither region showed theta modulation of HD cell spiking.

**Conclusions:**

That the cells can immediately respond to subtle differences in spatial landmarks is consistent with rapid processing of visual snapshots or scenes; similarities in PoS and RSC responding may be due either to similar computations being performed on the visual inputs, or to rapid sharing of information between these regions. More generally, this two-cue HD cell paradigm may be a useful method for testing rapid spontaneous visual discrimination capabilities in other experimental settings.

## Introduction

How the brain forms a representation of external, navigable space is a current area of intense enquiry because it involves transformation of sensory inputs into higher-order, more abstract cognitive structures, and thus has wide relevance to cognition generally. One of the foundations of the place representation is the ‘sense of direction’, supported by a network of brain regions known as the head direction (HD) system, which uses previously learned visual landmarks to re-orient when an animal re-enters a familiar environment. This study investigates the neural basis of this rapid orientation process, which is important for understanding how perception and memory processes shape the place representation.

The HD system in rodents (and probably all vertebrates) contains so-called HD cells, which fire when the animal faces in a particular direction, regardless of position, and are close to silent otherwise (Taube et al., 1990a; 1990b). Each cell has its own preferred firing direction (PFD) in a given environment, and the ensemble of HD cells is coherent – that is, the cells maintain the same relative PFDs, even if the orientation of the entire cell population changes from one environment to the next. Local environmental landmarks – mainly visual – establish the population orientation when the animal enters an environment, and the signal is updated as the animal moves around by means of self-motion information such as vestibular, optic flow, motor efference and proprioceptive cues to movement (Taube, 2007; Yoder et al., 2011). Although HD cells typically begin firing essentially immediately on entry into a familiar environment (Jankowski et al., 2014), the environmental cues are learned, not hard-wired (Taube and Burton, 1995), which means that some kind of rapid recognition process must take place. How and where this occurs is not known, but is likely to be in HD regions close to the visual system.

Current evidence suggests that the postsubiculum (PoS) and the retrosplenial cortex (RSC) have an important role in processing landmark information derived from visual areas and relaying this input to interconnected areas of the HD cell circuit and the hippocampal formation (for review, see Yoder et al., 2011). Consistent with this hypothesis, Goodridge and Taube (1997) found that tuning curve precision (width of the tuning curve) and stability of anterior thalamic HD signals were impaired after PoS lesions, and the cells showed reduced responsiveness to rotations of the landmarks. Yoder et al. (2015) reported a similar effect on lateral mammillary HD neurons, as did Calton et al. (2003) on CA1 place fields, downstream of the HD signal. Similarly, Clark et al. (2010) found the same effects on both tuning curve width/stability and landmark control following RSC lesions, although to a lesser extent. In contrast, lesions of the parietal (Calton et al., 2008) or the postrhinal (Peck and Taube, 2017) cortex did not impair landmark control of anterior dorsal thalamus (ADN) HD cells, suggesting that the processing of visual landmark information is routed via the PoS and the RSC. These studies collectively point to a likely role for PoS and RSC in passing information about landmarks (or at least their visual properties) to the subcortical circuits that collate the HD information and generate a stable signal. It is thus of interest to look for possible differences in the contributions made by the two regions.

**Figure 1.**
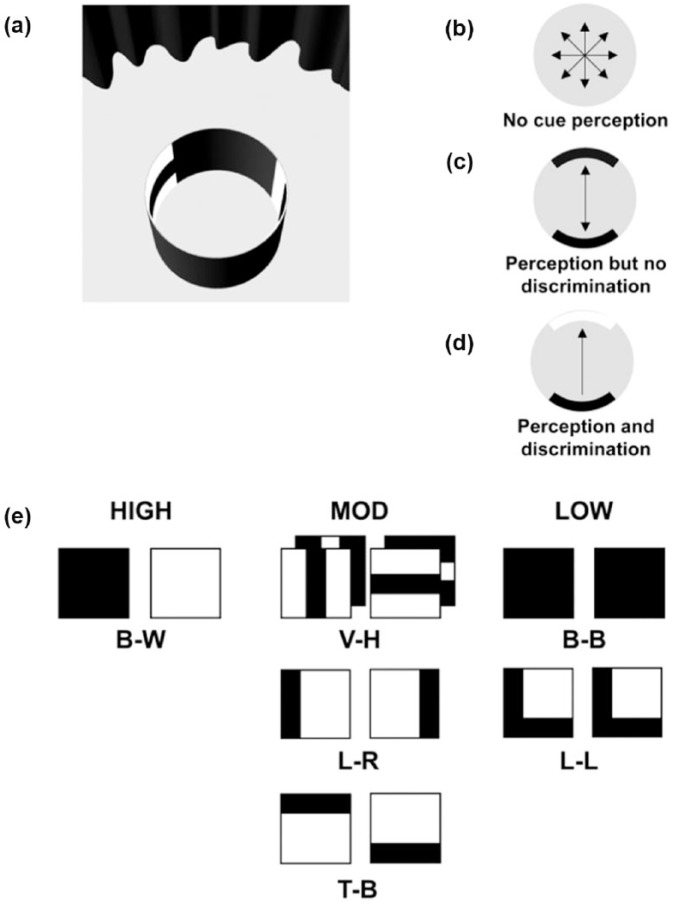
Cue-discrimination setup for recording HD cells in the RSC and PoS of freely moving rats. (a) Recording environment. Rats foraged for rice in a 50 cm high by 74 cm diameter cylindrical arena with two cue cards 50 × 50 cm attached to the inner wall 180° apart. The arena was situated in the centre of a 260 cm diameter, black circular curtained enclosure. (b)–(d) Environmental symmetry, and hence HD cell anchoring, varies as a function of cue perception. (b) If no cues are detected then the environment has infinite rotational symmetry, and HD orientations might be random. (c) If the cue pair is detected by the cells but not discriminated, then the environment has twofold rotational symmetry and an HD cell might fire in either of two directions, at random. (d) If the cues are both detected and discriminated, then the environment lacks rotational symmetry (is polarised) and an HD cell would be able to fire in a constant direction. (e) Cue card patterns, grouped into discriminability categories (high, moderate and low), and their corresponding abbreviations. B-W: black–white, V-H: vertical–horizontal, L-R: left–right, T-B: top–bottom, B-B: black–black and L-L are both L-shaped. The V-H cards were also sometimes used in white-on-black configuration.

It is not known why there should be two separate landmark processing regions, but one possibility is that they differ in some aspect of their contribution to the processing of environmental landmarks. We therefore set out to compare the responses of these neurons during re-orientation in a situation where detailed landmark-cue processing is required. We recorded HD neurons as rats foraged in a cylinder within a curtained enclosure (Figure 1(a)). The infinite rotational symmetry of this arrangement (Figure 1(b)) was broken by a pair of cue cards on the cylinder wall, located opposite each other; the resulting twofold symmetry of the cue pair (Figure 1(c)) was further broken by making the cards different so that the environment was now polarised, provided the cues could be discriminated (Figure 1(d)). The discriminability of the cue card pair varied between high, moderate and low (Figure 1(e)) – in the HIGH condition, one card was black and one white; in the MOD condition, a black bar was oriented and/or positioned one way on one card and the other way on the other, and in the LOW condition, the cards were visually identical (although they may have been distinguishable by smell; hence discriminability is assumed to be low and not zero).

PoS and RSC HD cells were recorded in sessions ranging from 4 to 12 trials; between trials, the rat was removed and mildly disoriented, and the entire environment was frequently also rotated to disconnect it from static external cues. Cells were tested for basic firing parameters (firing rate, spiking characteristics, and possible spatial localisation) and to determine whether their firing directions rotated appropriately with the cue pair, indicating successful detection and discrimination.

## Materials and methods

### Subjects

A total of 18 adult male Lister Hooded rats weighing between 317 and 437 g at the time of surgery were used for the experiments. One PoS rat had received a saline sham injection into the lateral geniculate nucleus (LGN), as part of a different study. The rats were housed in individual cages in a temperature and humidity-controlled colony room that was kept on an 11:11 h light:dark cycle plus 1 h each of half-light simulated dawn and dusk. All the rats had free access to food and water prior to surgery and began food restriction to maintain 90% of their free-feeding weight 1 week after surgery. All procedures were licensed by the UK Home Office following the revised ASPA regulations (2013) modified by the European Directive 2010/63/EU. Two of the RSC-implanted rats also took part in another experiment, in a different apparatus (Jacob et al., 2016).

### Electrodes and surgery

Each rat was anesthetised and implanted with a microdrive (Axona) that was configured with either four or eight tetrodes threaded inside a guide cannula. Each tetrode was made of individual 90:10 platinum–iridium wires (California Fine Wire) of diameter 25 µm (for the four-tetrode drives) or 17 µm diameter (for the eight-tetrode drives). The tips of the electrodes were plated in a 1:9 0.5% gelatine:Kohlrausch platinum solution to approximately 250 kΩ using a pulse generator (Thurlby Thandar TGP-110) and a current source amplifier (A.M.P.I. ISO-FLEX) that delivered 2 µA current for 550 ms to each channel. The microdrive and the guide cannula were fastened to the skull with dental acrylic (Simplex Rapid) covering seven supporting screws of 1.6 × 3 mm (Small Parts) that were inserted into the occipital, parietal and frontal cranial bones. One of the supporting screws made contact with the frontal cortex and was connected to the microdrive ground wire.

Implant coordinates were based on previous studies of HD cells in the RSC (Cho and Sharp, 2001) and the PoS (Taube et al., 1990a); for RSC (n = 12 rats; n = 6 left hemisphere, n = 6 right hemisphere), these were 5.4 mm posterior to bregma, 0.6–0.8 mm lateral to the midline and 0.2–0.9 mm ventral from the cortical surface, while two sets of coordinates were used for the PoS (n = 5 rats; all in the left hemisphere); being, respectively, 6.7 or 7.5 mm posterior to bregma, 2.8 or 3.2 mm lateral to the midline and 1.6 or 1.9 mm ventral from the cortical surface. After surgery, the animals were monitored until they awoke, and meloxicam (Metacam) was given in jelly for three consecutive days as pain relief. All animals were allowed to recover for 1 week prior to the start of recording.

### Cue control apparatus

The cue control experiments were performed inside a cylindrical arena (diameter, 74 cm; height, 50 cm) made of plywood painted with light grey matt acrylic and placed at the centre of a black curtained enclosure (diameter, 260 cm) (Figure 1(a)). A cylinder was selected as a recording arena to minimise the influence of the environment’s geometry as an orienting cue (Golob et al., 2001; Knight et al., 2011). Attached to the inner wall of the cylinder with Velcro tape were two 50 × 50 cm cue cards (Figure 1(b)) made from black and/or white polypropylene sheets, each subtending ~77° of arc, and located 180° apart. Two of the cue pairs were plain – either both black (identical-cue controls; B-B) or one black and one white (a maximally salient high-contrast condition; B-W). The remaining cue pairs, with one exception, were made from white card decorated with a black bar of 14 × 50 cm; these were thus equal in overall luminance and contrast, so discrimination would require processing of the cues’ internal structure. The position of the bar in each pair differed in, respectively, orientation (vertical vs horizontal; V-H cues), lateral position (left vs right; L-R cues) or vertical position (top vs bottom; T-B cues). For the exceptional pair, black and white were reversed for the orientation cues.

The arena rested over a black vinyl sheet and was lit from above by six light fixtures that provided approximately 250 lux of light, with a radio attached to the ceiling as a source of white noise to reduce the effect of directional auditory cues.

### Screening/recording procedures

#### Signal processing and tracking

Single units were recorded using a headstage amplifier connected to a microdrive and connected to the multichannel recording system (DacqUSB, Axona) via a flexible lightweight tether. Traces from individual channels were collected at a sampling rate of 48 kHz, amplified 6000–20,000 times and band-pass filtered from 300 Hz to 7 kHz. The traces from each electrode were referenced against an electrode from a different tetrode that showed low spiking activity. Spikes were defined as short-lasting events that crossed a user-defined threshold; the period from 200 µs before to 800 µs after threshold-crossing were captured and saved along with the corresponding timestamps. To track the rat’s head position and facing direction in the horizontal plane, two arrays of light-emitting diodes (LEDs), one large and one small, were transversely positioned 8 cm apart on a stalk connected to the headstage. The LED positions were recorded at a sampling rate of 50 Hz by a camera attached to the ceiling. Spike times, the rat’s head position in x- and y-coordinates and its heading in degrees were saved for offline analysis.

#### HD cell screening

The screening sessions for single units tuned to HD were conducted inside a 76 × 76 × 50 cm box in a room separate from where the cue control experiments took place. The animals had visual access to a polarising cue card placed inside the box, and distal room cues. The screening sessions consisted of 5–10 min trials during which the rat foraged for rice while spikes were monitored. Polar plots were generated with spike-sorted data using the software TINT (Axona) and examined to determine whether single units were tuned to HD. In screening sessions during which HD cells were not found, the tetrodes were lowered by ~50 µm and the rat was screened again 4 h later or the next day.

#### Cue control procedure

After isolating single units tuned to HD during screening, the rat was taken to the recording room inside a closed opaque box. A cue control session was conducted using a similar recording procedure as described previously (Knight et al., 2011). Briefly, each session started with a series of baseline trials (2–4 trials) where the two cue cards remained aligned in the same location relative to the room coordinates. This was followed by a series of rotation trials (4–8 trials) during which the cue cards were rotated together by ±45°, ±90°, ±135° or 180° (Figure 1(c)) to test whether HD cells could discriminate and use the visual features of the cue cards as orienting landmarks (Figure 1(d)). For each recording session, the starting location of the cue cards as well as the magnitude and direction of the cue rotations were pseudo-randomised. The length of each trial was 300 s and was initiated via remote control after placing the rat inside the cylinder with a pseudo-random location and facing direction. Prior to each trial, the cue cards, recording arena and the base of the cylinder were inverted and wiped with 75% ethanol to scramble olfactory cues. During the inter-trial interval, the rats remained inside a holding box outside the curtained enclosure, and then prior to being replaced in the recording box they were mildly disoriented by being passively transported and rotated in the holding box around the periphery of the curtained enclosure, thereby preventing the animals from using self-motion cues to track their orientation. During the recording trials, the experimenter remained outside the curtained enclosure, tossing rice into the arena to encourage the rat to sample all the locations and facing directions within the cylinder. With the exception of the black–white (B-W) cue pair, which was used to establish cue control, the order of presentation for the different cue card stimuli was pseudo-randomised between rats to prevent temporal effects due to the changing experience of the animal across days.

For one rat, a control procedure was conducted to assess the relative contribution of vision and olfaction to the landmark discrimination. Using the patterned cues that had rotational symmetry (V-H, T-B and L-R), the visual identity of the cards was reversed by rotating around their central points, 90° for V-H and 180° for T-B and L-R, to determine whether firing remained aligned relative to the physical cards or to the visual appearance.

### Data analysis

Spike-sorting was performed using KlustaKwik (Kadir et al., 2014) followed by manual refinement using the TINT software package (Axona). Polar plots were generated, and cells that showed directional firing by eye were selected for further quantitative analysis, which included extracting peak firing rate and directional clustering – Rayleigh vector (R-vector) – scores. Cells that were recorded on the same tetrode across sessions were treated as unique if more than 5 days separated the sessions. For each trial, a cell was accepted into further analysis if it had a peak firing rate >1.0 Hz across sessions, a peak:mean rate ratio > 2 and a R-vector p-value <0.05. Cells were excluded altogether if only three trials or fewer per session remained after selection.

Data were grouped in three ways: (a) Those pertaining to cell-specific characteristics, such as firing rate, R-vector score and tuning curve width were averaged across a given session. (b) Data concerning trial-specific characteristics such as firing direction relative to the cue card array were averaged across a given cell. (c) Data concerning session-specific characteristics, such as the spread of firing directions, were averaged across a session.

**Figure 2.**
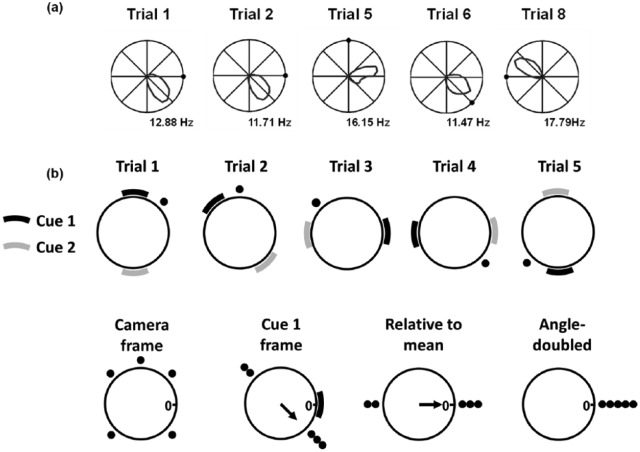
Tuning curve analysis. (a) Example of the tuning curve derived from a single PoS HD neuron in a series of recording trials, selected from a 12-trial session. The plots show firing rate as a function of HD, normalised to the peak rate (shown). (b) Schematic from a hypothetical set of trials showing how data were transformed into a common reference frame to allow population comparisons. The original reference frame was that of the room-fixed camera: the top row shows an idealised trial in which the cell consistently rotated its firing with the cue pair, but was aligned relative to Cue 1 on trials 1, 2 and 5 and 180° away from it on trials 3 and 4. The bottom row shows these data expressed in the different reference frames: in the camera frame, the firing direction is scattered; relative to Cue 1, it is bipolar with three trials in one direction and two in the opposing direction; relative to the session mean (arrow), the bulk of the firing is now aligned to zero, and after angle-doubling the bipolar distribution is now transformed to unipolar.

The basic protocol for firing direction analysis is shown in Figure 2. First, a tuning curve was derived (see below). Each tuning curve was recorded in the camera frame of reference, within which the cues rotated, but it was necessary to align these within a common reference frame so that population statistics could be derived. To do this, the tuning curves were first specified relative to one of the two cues (‘Cue 1’; Figure 2(b)) and the mean firing direction across the session computed. Then, the tuning curves were realigned relative to this mean; this value (deviation from the session mean) became the value that entered into the population analysis. Finally, the angles were doubled in order to remove any bipolarity that might be present due to cue confusion – this was done so as to enable determination of cue use independently of cue discrimination.

### Cell-specific firing characteristics

Data were analysed using MATLAB (MathWorks) with custom-made programs and functions taken from the CircStat toolbox (Berens, 2009). To analyse the firing characteristics of single units, the spike times and position samples of the rat’s facing direction were sorted into bins of 6°. The mean firing rate per HD bin (Hz) was calculated by taking the sum of the spikes divided by the total amount of time (s) that the rat’s facing direction was located in each bin. A 5-bin (30°) smoothing kernel was applied to the circular histogram of the firing rate as a function of HD to minimise random influences to the firing rate in each cell (Abeles, 1982). Polar plots of the smoothed histograms were generated to visualise the cell’s tuning curve (Figure 2(a)).

The parameters that were used to quantify the tuning curve characteristics for each cell were firing rate, PFD, tuning width, and mean R-vector length. The firing rate of the HD cell was defined as the bin with the maximum firing rate; that is, the mode of the circular histogram, and the PFD defined to be the directional bin of the mode. The tuning width, or directional firing range of the cell, was determined by computing two standard deviations from the circular mean direction. The R-vector score was used as a measure of the directional tuning in each cell. Values of R close to 1 indicate that the spikes are closely clustered around a single value and values close to 0 indicate that the spikes were distributed around all facing directions.

To examine how a cell behaved in relation to the cue card pair across trials, the PFDs were realigned according to their relationship to one of the two cues (arbitrarily called ‘Cue 1’) and the circular mean of the session values was computed. Each PFD was then expressed as a deviation from this mean.

In some cases, it was necessary to test whether a low R-vector score could be due to bipolarity of the firing distribution. To do this, the values were doubled and plotted modulo 360 – this has the effect of wrapping the 180° points around to zero, thus rendering a bipolar distribution unipolar for the purposes of analysis.

#### Test for bipolar tuning curves

In order to test whether there was any within-trial tendency towards bipolarity, due to (perhaps) intermittent reversing of the tuning curve arising from confusion between the cues, a test for bipolarity was conducted by running a circular autocorrelation on the smoothed binned data from each trial, using the MATLAB circshift and corr functions. From the autocorrelation, the values at 90° and 270° relative to the peak were extracted and averaged, and compared with the peak at 180° using a one-tailed t-test of the hypothesis that the 180° peak would be larger.

#### Spatial firing patterns

In order to determine whether there was any spatial specificity to the firing, which could be of computational utility (Bicanski and Burgess, 2016), and has been reported for some types of HD cells in cortical regions (Cacucci et al., 2004; Peyrache et al., 2016), we examined the spatial distribution of spikes in the same way as is usually done for place cells, extracting the spatial information content of the firing, the coverage relative to the whole environment and the coherence of the firing distribution. Path data were extracted by smoothing the position points with a 400 ms window and first plotting the spikes at their corresponding locations, for visual inspection. These data were then used to generate dwell-time-normalised firing rate maps by binning the spike and position data into 3 cm bins and dividing the firing rate by dwell time, and then smoothing the map with a boxcar of three spatial bins (whereby the value in each bin was replaced by the average for that bin plus the surrounding eight bins). To eliminate sampling bias due to the rat’s inability to face all directions everywhere in the arena, we undertook the spatial analysis using only the inner 50% of the arena, in which directional sampling was homogeneous. In case some cells might have place fields near the edge of the arena, we also analysed the hemi-cylinder lying in the direction of the cell’s PFD, for which the directional bias was much reduced (since it was easy for the rat to face in the cell’s PFD in this region); this made little difference, so we chose the inner region as being the most conservative measure.

Spatial information in bits/spike was computed following the method of Skaggs et al. (1993). Coverage was calculated as the percentage bins above 20% peak rate, and coherence of spatial firing was determined using a Pearson’s correlation between the smoothed and unsmoothed rate maps. Actual data were compared against a control dataset generated by taking the spike data and shifting it forwards by 1000 position points (20 s), which would scramble any spatial specificity of firing while leaving the temporal dynamics unchanged. Each firing rate map was compared with its shuffled control map using a paired t-test.

#### Temporal firing patterns

We looked at temporal patterns of firing including inter-spike interval (ISI) time to peak and decay time to half-peak, as well as theta-frequency rhythmicity. For the ISI analysis, only trials with >145 spikes were used and only one trial (usually the first) was used from each cell. A histogram of ISIs with 2 ms bins was generated for each cell and the peak was taken as the centre of the bin with the highest count. We calculated decay time by fitting, to the histogram, a one-term exponential decay function of the form $y=a^{bx}$ from the peak to peak + 1 s, using the fit function from MATLAB’s Curve Fitting toolbox. Time to half-peak was then taken as the time taken for the exponential fit to decay to half the peak value.

We measured theta modulation by plotting autocorrelograms of the spike trains over the range ±500 ms, in bins of 10 ms duration. The plots were then highly smoothed (20 bins) to remove local variations, and the values at the 7th bin from the central peak (expected trough at 60–70 ms) and the 12th bin (expected peak at 120–130 ms) determined: the theta modulation index was taken as the difference between these values divided by their sum. If there is significant theta modulation, then the 12th bin should be a peak and the 7th bin a trough, yielding a positive modulation index varying from 0 to 1. Conversely, values below zero would indicate a descending likelihood of a cell spiking with time between the first and second time-points.

#### Movement correlates

The relationship between linear or angular speed and firing rate was examined by analysing those portions of the trial when the animal’s HD was within 45° either side of the PFD of the cell, and correlating the firing rate with movement speed. Correlations of firing rate with linear running speed and angular head velocity (AHV) were computed as percentage firing rate change as a function of movement speed, to compensate for variability in firing rate between cells. Running speeds below 2 cm/s were excluded from running speed analysis. Linear running speed was binned in intervals of 2 cm/s, and AHV was binned in intervals of 2°/s. The firing rate was calculated by counting the spikes in each bin and dividing by the time spent in that bin (dwell time) and then normalised to the peak for that trial to enable comparison across trials/cells. Bins with dwell times of less than 1.5% total trial time or with fewer than five spikes were discarded; a linear regression was run on the remainder to generate a slope value. Because dwell time decreased with increasing velocity, which might cause artefacts in the rate/speed relationship, the baseline for each trial was calculated by generating an artificial continuous 10 Hz spike train, analysing it in the same way and subtracting this control slope from the raw data slope. For AHV, left and right turns were analysed separately and the absolute slope values then combined for that cell. This is because previous recordings from other brain regions have found cells with asymmetric AHV rate profiles (Bassett and Taube, 2001), being negative in one direction and positive in the other, which would cancel if the raw values were taken. The resulting difference values were entered into a t-test comparing PoS and RSC.

Anticipatory time intervals (ATIs) were estimated using a time slide analysis in the manner of Blair and Sharp (1995). Given the camera sample rate of 50 Hz, spike times were shifted forwards in 20 ms intervals from 20 to 160 ms. For each head turn, a tuning curve was constructed for leftwards and rightwards head turns in the manner described. A population vector method was used to calculate the PFD of each tuning curve (Song and Wang, 2005) as

$$PFD=\arctan\left(\frac{\sum_{i}^{N}r_{i}\sin(x_{i})}{\sum_{i}^{N}r_{i}\cos(x_{i})}\right)$$

where *r_i_* is the firing rate in bin *i* of *N* with mean bin direction *x_i_*, and arctan denotes quadrant-specific arctangent function.

The difference between PFD for leftwards and rightwards tuning curves was plotted, and a line was fitted to it using the MATLAB function polyfit. To allow for estimations of ATIs at a finer scale than measurement, the ATI was taken as the value of this fitted line when PFD difference was equal to zero, extracted using the MATLAB function polyval.

### Stabilisation analysis

To investigate the time course of cue control establishment, we looked at firing within the PFD range (PFD ±20°) across the trial, taking both the percentage of total spikes emitted in each time decile (30 s – trials were 300 s long), and the time taken to reach each spike count decile.

#### Histology

After completion of the electrophysiological recordings, the rats were anaesthetised and killed with an overdose of sodium pentobarbital Euthatal, 150 mg/kg and perfused transcardially with saline followed by 4% formalin solution. A day before sectioning, the brains were placed in 4% formalin/20% sucrose solution for cryoprotection. Coronal or sagittal sections of the brains were cut in 40 µm sections using a microtome at −20° C and mounted on microscopic slides (Superfrost BDH). The brain sections were Nissl stained with 0.1% cresyl violet (Sigma-Aldrich) or 0.5% thionin (Sigma-Aldrich) and cover-slipped with DPX (Sigma-Aldrich). The slides were examined under a light microscope (Leica) and imaged with a digital camera mounted on the microscope. Images with visible electrode tracks were saved for histological analysis. The electrode location was verified by examining the brain region where the final electrode track was located and then relating the location to a standard rat atlas of the regions (Paxinos and Watson, 2007).

## Results

Representative example sections showing the final tetrode placement of rats implanted in the PoS and the RSC are shown in Supplementary Figure 1. A total of 149 unique cells meeting the criteria for HD cells (see section ‘Methods’) were recorded from 18 rats in 78 sessions, of which 74 cells were recorded from 6 rats implanted in the PoS over 34 sessions, and 75 cells from 12 rats implanted in the RSC over 44 sessions. We observed that a greater proportion of RSC HD cells (73%) were found below 1500 µm which is likely in the deeper granular region. However, although the implant target coordinates were the same for all rats, there was a degree of variability in the actual anterior-posterior and medio-lateral coordinates in which the tetrodes were implanted (Supplementary Figure 1). Furthermore, the tetrodes did not cover all the layers of the granular RSC as illustrated by the final tetrode depth, which could have under-estimated the actual number of HD cells.

Results and statistics are detailed below and summarised in Table 1.

**Table 1.** Basic firing statistics compared between PoS and RSC HD cells.

	PoS (n = 74)	RSC (n = 75)	F/T statistic (dof)	p value
Peak rate (Hz)	8.14 ± 0.63	22.40 ± 2.58	t(147) = 5.34	3.48 × 10^−7^
Mean rate (Hz)	1.83 ± 0.19	5.07 ± 0.52	t(147) = 5.86	2.92 × 10^−8^
Tuning curve width (°)	43.65 ± 1.13	49.29 ± 1.28	t(147) = 3.31	1.18 × 10^−5^
Tuning curve R-vector	0.59 ± 0.02	0.53 ± 0.02	t(147) = 1.53	0.13
Spatial information (data: control bits/spike ratio)	1.63 ± 0.06	2.22 ± 0.10	t(145) = 5.00	7.00 × 10^−7^
Coverage (data: control % ratio)	0.98 ± 0.01	1.48 ± 0.04	t(145) = 3.67	1.70 × 10^−4^
ISI rise time to peak (ms)	13.5 ± 1.6	7.5 ± 0.4	t(144) = 3.74	2.66 × 10^−4^
ISI decay time to half-peak (ms)	80.1 ± 6.5	48.8 ± 5.8	t(144) = 3.61	4.20 × 10^−4^
Theta modulation index	−0.05 ± 0.03	−0.03 ± 0.00	t(148) = 2.64	0.009
Abs. slope of firing rate correlation with linear running speed (%/m/s) vs 10 Hz control	0.69 ± 0.1 vs 0.06 ± 0.001	0.6 ± 0.1 vs 0.07 ± 0.01	Data vs 10 Hz control F(1, 1) = 40.10	< 0.0001
			PoS vs RSC F(1, 1) = 0.50	0.50
			Interaction F(1, 64) = 0.30	0.56
Abs. slope of firing rate correlation with angular head velocity (%/°/s) vs 10 Hz control	0.12 ± 0.01 vs 0.03 ± 0.05	0.08 ± 0.01 vs 0.03 ± 0.03	Data vs 10 Hz control F(1, 1) = 9.70	0.003
			PoS vs RSC F(1, 1) = 5.0	0.003
			Interaction F(1, 64) = 4.20	0.025
Anticipatory time interval (ms)	14.37 ± 8.19	47.91 ± 4.28	t(147) = 3.62	0.0002

PoS: postsubiculum; RSC: retrosplenial cortex; dof: degrees of freedom: ISI: inter-spike interval.

N’s differ slightly because some of the data trials could not be curve-fitted.

### Cell-specific firing parameters

As described in section ‘Methods’, the cell-specific characteristics of peak and mean firing rate, R-vector score and tuning curve width were calculated for each trial and then averaged across a given session for each cell. Statistical parameters are summarised in Table 1 and graphically shown in Supplementary Figure 2(A). Peak firing rates were significantly higher in RSC, being 8.14 ± 0.63 Hz in PoS and 22.40 ± 2.58 Hz in RSC (t(147) = 5.34, p = 3.48 × 10^−7^). Similarly, mean firing rates were higher in RSC: 1.83 ± 0.19 in PoS and 5.07 ± 0.52 in RSC (t(147) = 5.86, p = 2.92 × 10^−8^). Tuning curves were narrower in PoS, being 43.65° ± 1.13° in PoS and 49.29° ± 1.28° in RSC (t(147) 3.31, p = 1.18 × 10^−5^). However, directional tuning was similar in both structures: there was no difference in R-vector score, being 0.59 ± 0.02 in PoS and 0.53 ± 0.02 in RSC (t(147) = 1.53, p = 0.13).

### Spatial firing

Visual inspection of the firing rate maps revealed mostly uniform firing, although there were sometimes patches of inhomogeneous firing at a single-trial level and occasional clear place fields of the kind seen in hippocampal recordings (Supplementary Figure 3). We quantified spatial modulation by considering spatial information content, coverage and coherence. As detailed in section ‘Methods’, only the central 50% (by radius) of the arena was used in order to remove bias induced by inhomogeneous directional sampling around the arena edges.

Both PoS and RSC HD neurons showed evidence of a spatial component to their firing, revealed by higher spatial information content, lower coverage and by higher coherence relative to the time-shifted control data (Supplementary Figure 2(b)). Spatial information content (bits per spike) for the PoS data was 0.23 ± 0.02, while for the control data it was 0.16 ± 0.01, which was significantly different, as revealed by a paired one-tailed t-test (t(73) = 11.57, p = 9.72 × 10^−18^). Corresponding values for the RSC data were 0.15 ± 0.01 and for the control data were 0.07 ± 0.00, which was significantly different (t(73) = 11.16, p = 1.13 × 10^−17^). Coverage was slightly less for both cell types in the real data; PoS coverage in the real data condition was 52 ± 3%, and in the time-shifted control condition was 53 ± 3% which was significantly greater (paired one-tailed t(72) = 4.14, p = 4.70 × 10^−5^). For RSC, coverage for the data was 62 ± 2% and for the control data was 64 ± 2%, which was also significantly higher (t(73) = 3.58, p = 3.1 × 10^−4^). Coherence values were greater for the data compared with the time-shifted control data. For the PoS data were 0.24 ± 0.01 and for the time-shifted control data were 0.20 ± 0.01, which was significantly lower (t(70) = 6.38, p = 8.4 × 10^−9^), while for RSC the real data had a coherence value of 0.27 ± 0.01 and the control data had a coherence of 0.19 ± 0.00, which was again significantly lower (t(73) = 12.01, p = 2.89 × 10^−19^).

We then compared spatial firing bias between PoS and RSC by computing the ratio of data to control values for each session and then comparing between cell types for each of the three spatial parameters. For spatial information, the ratio of data: control was 1.63 ± 0.06 for PoS and 2.22 ± 0.10 for RSC, which was significantly different (t(145) = 5.0, p = 7 × 10^−7^, d = 0.83). For coverage, the ratio for PoS was 0.98 ± .01 and for RSC was 0.99 ± .01, which did not differ (t(145) = 0.67, p = 0.25). For coherence, the ratio for PoS was 1.26 ± 0.04, and for RSC it was 1.48 ± 0.04, which was significantly different (t(145) = 3.67, p = 1.7 × 10^−4^, d = 0.61).

Overall, then, both PoS and RSC HD neurons showed a small amount of spatiality to their firing when compared with a time-shifted version of the same data, having higher spatial information and spatial coherence; this was more pronounced in RSC. This accords with the visual inspection showing occasional and reproducible spatial inhomogeneity of firing. It thus appears that there is a degree of spatial modulation of firing, but this was rather slight. For RSC, this is surprising given previous reports of conjunctive spatial and directional firing in this structure (Cho and Sharp, 2001). However, it is consistent with our previous studies of RSC HD cells, in which we have observed very little clear spatial firing (Jacob et al., 2016; Knight et al., 2014).

### Temporal components of firing

To analyse temporal patterns of spiking, we took one trial (usually the first) for each cell and used the ISI histogram (Supplementary Figure 4) to determine the typical time between spikes (ISI peak) and spread of firing intervals (decay time to half-peak; Table 1). Consistent with our observations of higher firing rates in RSC, the time to the ISI peak was considerably longer in PoS (13.5 ± 1.6 ms) than in RSC (7.5 ± 0.4 ms), these being significantly different (t(144) = 3.74, p = 2.66 × 10^−4^). The time to return to the half-peak, which can be thought of as a measure of the prevalence of longer ISIs, was also different, being 80.1 ± 6.5 ms for PoS and 48.8 ± 5.8 ms for RSC (t(144) = 3.61, p = 4.2 × 10^−4^).

For each cell, autocorrelograms were generated for each trial in a session (Supplementary Figure 5(A)) and from these, a theta modulation index was calculated (see section ‘Methods’). Visual inspection of the autocorrelograms revealed no evidence of theta modulation, although there were frequently peaks and troughs suggestive of slightly longer scale periodicity (see examples from the cell shown in the Supplementary Figure 5(A)). However, these patterns did not persist across trials and are likely due to the dynamics of the animal’s movements. For example, if a rat is foraging by sweeping its head back and forth, then a cell’s tuning curve will be visited and re-visited over a regular time period of up to several seconds. It may be that rats varied their movement patterns across trials.

The quantitative theta modulation index confirmed the visual impression: in general, values were, if anything, negative (a lower value at the expected peak than at the expected trough) and slightly more so, and more dispersed, for PoS (Supplementary Figure 5(B)). The values for PoS were −0.049 ± 0.006 and for RSC were −0.032 ± 0.002 (t(148) = 2.63, p = 0.005).

### Movement correlates

Correlations of firing rate with linear running speed and AHV were computed as described in section ‘Methods’, yielding percentage firing rate change as a function of movement speed. For linear running speed, there was a weak correlation of firing rate with running speed relative to the control 10 Hz spike train; this was 0.69 ± 0.1%/m/s for PoS and 0.61 ± 0.1%/m/s for RSC. A two-way analysis of variance (ANOVA) of data type (real vs 10 Hz control) and brain area found a main effect of data (F(1, 1) = 40.1, p < 0.0001), but no effect of brain area (F(1, 1) = 0.50, p = 0.5) and no interaction (F(1, 64) = 0.30, p = 0.56).

**Figure 3.**
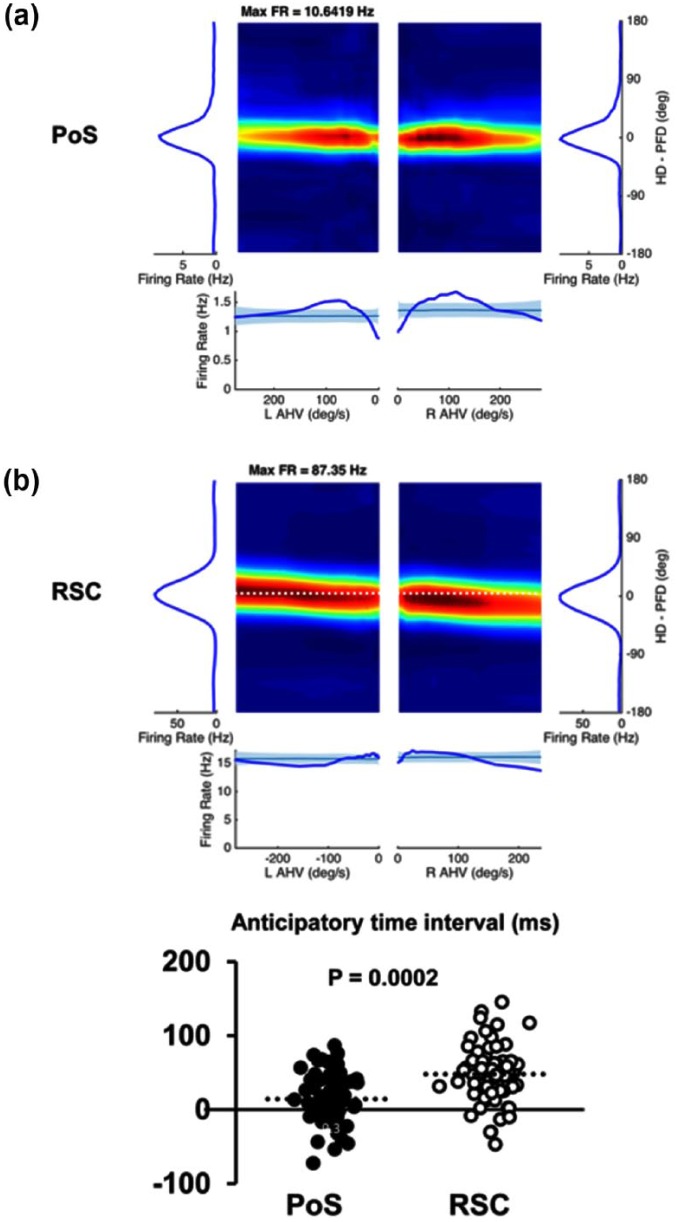
Modulation of HD cell firing by angular head velocity (AHV). (a) The heat plots show firing rate (colour; max = red) as a function of HD (y-axis) and AHV (x-axis) for a PoS and RSC neuron. Tuning curves collapsed across all AHVs for left and right head turns are shown to the left and right sides, respectively. Firing rate collapsed across HD as a function AHV is shown below each plot (means and standard error of the mean (SEM) of the shuffled control shown in blue). The left–right downwards slope evident in the RSC plot reveals anticipatory firing, in which the tuning curve shifts in the positive direction (right) for left head turns and in the other direction for right head turns. The shift increased linearly with AHV, revealing a constant time lag. (b) Overall, anticipatory firing differed between PoS and RSC (dotted line shows mean).

For AHV, there was also overall a very weak relationship with firing rate relative to the control steady 10 Hz spike train; this was 0.12 ± 0.01%/°/s for PoS and 0.08 ± 0.01%/°/s for RSC. These effects, though small, were significant: a two-way ANOVA comparing data versus control slopes for PoS and RSC found a main effect of data type (F(1, 1) = 9.70, p = 0.003), a main effect of brain area (F(1, 1) 5.00, p = 0.03) and a significant interaction (F(1, 64) = 4.20, p = 0.025). Plots of firing rate against HD and AHV (Figure 3) revealed an additional difference between PoS and RSC neurons. An example of each (the closest cell to the mean in each case) is shown in Figure 3(a), where it can be seen that the cell’s directional tuning deviates as a function of AHV, with greater deviation for higher AHVs in the RSC but not the PoS neuron. This deviation reflects anticipatory firing, first reported by Sharp and colleagues for anterior thalamic HD neurons but not PoS (Blair et al., 1997; Blair and Sharp, 1995) and replicated by Taube and Muller (1998); anticipatory firing was subsequently shown to a lesser extent for RSC HD neurons (Cho and Sharp, 2001). It is determined here by taking the slope of the AHV/PFD relationship. Comparing PoS and RSC neurons (Figure 3(b)), we found that, as in previous studies, RSC neurons showed a greater ATI (47.91 ± 4.28 ms) than did PoS neurons (14.37 ± 8.19 ms); these were significantly different (one-tailed t(147) = 3.62, p = 0.0002). The PoS values did not differ from zero (t(74) = 1.75, p = 0.0836).

## Cue control

The next set of analyses investigated ensemble behaviour and aimed to determine the extent to which the cells were controlled by the visual cues: for these analyses, behaviour of the individual co-recorded cells in a trial was averaged to yield an overall value.

### Overall cue control

We first looked at whether there was overall cue control by the cue pair, irrespective of the visual stimuli feature, and whether this would differ for brain region or cue type. As described in section ‘Methods’ (Figure 2), the firing directions for each trial in a session were extracted and re-oriented relative to the session mean, so as to express them all in the same reference frame. Then, because visual inspection suggested that firing directions were sometimes distributed in a bipolar fashion, we doubled the angles modulo 360 and re-computed the firing directions; this has the effect of wrapping points at 180° around to 0/360°, and rendering a bipolar distribution unipolar. The angle-doubled data were compared against a control, shuffled dataset in which the PFD for each trial was randomly generated; this procedure was repeated until 1000 such pseudo-sessions had been obtained.

Overall, the 2140 cells × trials (149 cells in multiple trials) comprised 221 cells × sessions, which collapsed (after averaging across cells) to 113 sessions, of which 41 were from PoS and 72 from RSC. Overall, the number of cells recorded in each cue condition were 48, 166 and 17 for HIGH, MOD and LOW cues, respectively. The shuffle control procedure yielded R-vector scores of 0.31 ± 0.01 for both single and doubled values, so this was used as the threshold against which to statistically evaluate cue control.

**Figure 4.**
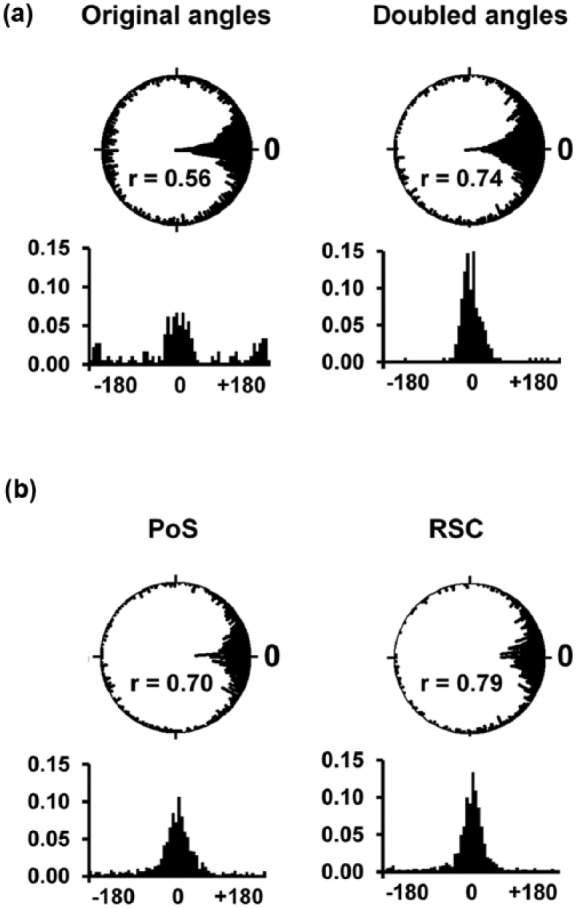
Overall cue control. The circular dot plots show the raw cell data, expressed relative to session means (0°); the histograms below these show the same data binned in 6° bins, linearised (centred on 0°) and expressed as a proportion of the total cell count in order to enable easier visual comparison between the cue types. (a) Angle-singled or doubled mean firing directions relative to the cue pair, for the individual trials. For the original angles, each cell’s preferred firing direction (PFD) was computed relative to Cue 1, and mean values computed for each session, each PFD was then realigned to this session mean (0°). For the doubled angles, the PFD value relative to the cue was doubled, modulo 360°, so as to wrap values near 180° around to zero and thus remove the bimodality. (b) The doubled-angled values compared between PoS and RSC for all trials. Statistics reported in the text were calculated using the session means rather than the set of individual trials.

The population of all firing directions before and after angle-doubling are shown in Figure 4(a). R-vector scores showed a marked increase after angle-doubling, from 0.56 to 0.74, indicating a degree of bipolarity in the original firing directions, therefore to evaluate overall cue control as a function of brain area, the angle-doubled values were used. Overall, the angle-doubled R-vector scores far exceeded the shuffled control threshold (t(112) = 23.00, p < 0.0001), indicating a high degree of cue-following. Average per-session R-vector scores for PoS were 0.73 ± 0.04 and for RSC were 0.79 ± 0.02; these values did not differ (two-tailed t(111) = 1.42, p = 0.16), indicating no difference between brain areas in the overall level of cue control (Figure 4(b)).

### Cue discrimination

**Figure 5.**
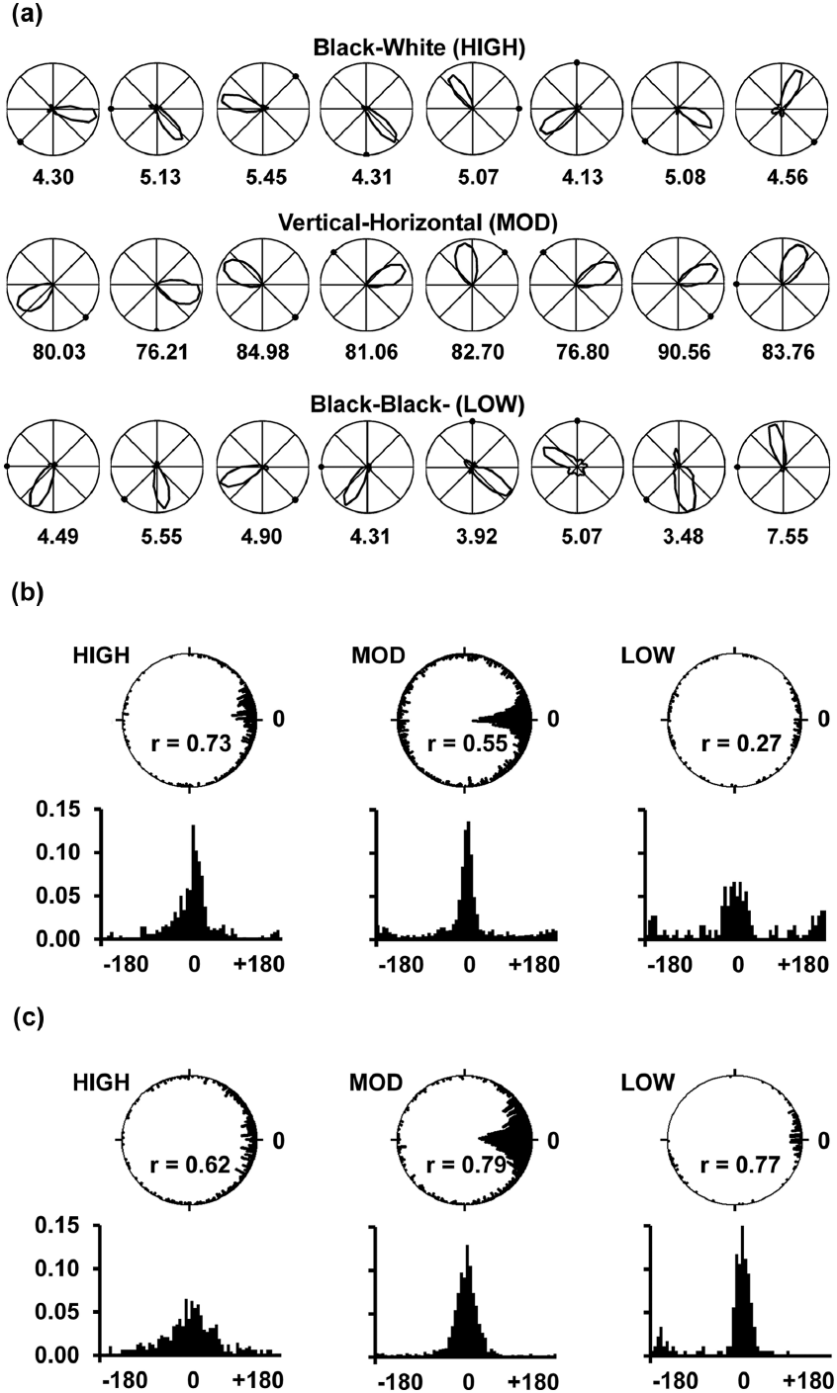
Comparison of responding to the three categories of visual cues. Dot plots and histograms are generated as for Figure 4. (a) Examples of eight recording trials from three HD cells, one from each cue condition, showing the relationship of firing direction (polar plot) to the index cue (black dot). Peak firing in Hz is shown below each plot. The first two cells are from RSC, the last from PoS. The cell in the HIGH discriminability condition showed much less variability, as was typical. (b) and (c) Population data. PoS and RSC data have been combined. (b) The original angles; (c) the doubled angles, showing increased dispersion for the highly discriminable cues (HIGH) but decreased dispersion for the MOD and LOW cues, indicating that their original firing directions had a degree of bipolarity. See text for statistical comparisons.

We next looked at cue discrimination as a function of cue type, first using the original non-doubled angles. If the cells were confusing cues, then the firing should be bipolar and the R-vector scores low. Indeed, firing directions (Figure 5) were more clustered for the more discriminable cues. R-vector scores were much higher for the HIGH cues (0.73 ± 0.05) than for the MOD cues (0.55 ± 0.03) or the LOW cues (0.27 ± 0.08). Unbalanced one-way ANOVA found this difference to be highly significant (F(2, 110) = 8.0, p = 0.0006), and post hoc testing (Tukey’s honest significant difference (HSD)) found that the HIGH cues had significantly higher R-vector scores than both the MOD cues (t(101) = 2.18, p = 0.03) and the LOW cues (t(23) = 6.58, p = 0.0001), while the MOD and LOW cues also differed (t(96) = 2.90, p = 0.005).

In order to determine whether the differences might be due to differential confusion between cues, we repeated the analysis using the angle-doubled values to remove bipolarity. R-vector scores for the HIGH cues were 0.62 ± 0.06, for the MOD cues were 0.79 ± 0.04 and for the LOW cues were 0.77 ± 0.23. ANOVA showed that scores for the HIGH cues were now significantly lower than for the MOD cues (t(101) = 3.01, p = 0.003) and not different for the LOW cues (t(23) = 1.63, p = 0.12); nor did the MOD and LOW cues differ (t(96) = 0.36, p = 0.72). Thus, the superior single-angle R-vector scores of the HIGH cues can be attributed to the more bipolar distribution of the MOD and LOW cues.

**Figure 6.**
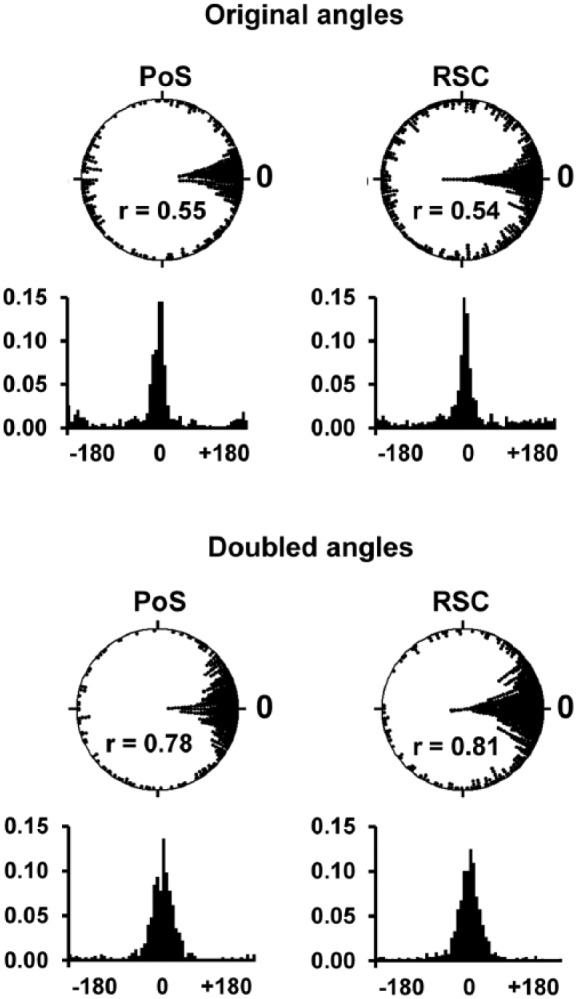
Comparison of responding to the MOD cues. Preferred firing directions were derived and plotted, together with R-vector score and normalised histograms, as for Figure 5. There was an improvement in score with the doubled-angle values, indicating a degree of cue confusion, but no difference in cue control between PoS and RSC (see text for details).

To compare between brain regions, we then looked in more detail at responses to the MOD cues, as these were the ones that were in principle discriminable but in practice evoked some confusion, suggesting maximal demand on landmark discrimination processing. However, we found no difference. The overall single- and doubled-angle data for all the MOD trials are shown in Figure 6, as a function of brain area. Comparison of the three cue types (R-L, T-B and V-H) yielded no differences in resulting R-vector score (F(2, 85) = 0.90, p = 0.39) so the data were analysed together. Basic single-angled R-vector scores for the two brain areas did not differ (PoS = 0.57 ± 0.05, RSC = 0.55 ± 0.04, t(85) = 0.23, p = 0.82). Similarly, although the doubled-angle scores significantly improved overall, from 0.55 ± 0.03 to 0.79 ± 0.02 (one-tailed paired t(87) = 7.13, p = 1.37 × 10^−10^), the doubled-angle Rayleigh scores were not different (t(85) = 0.33, p = 0.74). Thus, there was no difference in the degree of cue control versus cue confusion between brain areas.

### Comparison of visual and non-visual contributions to cue discrimination

**Figure 7.**
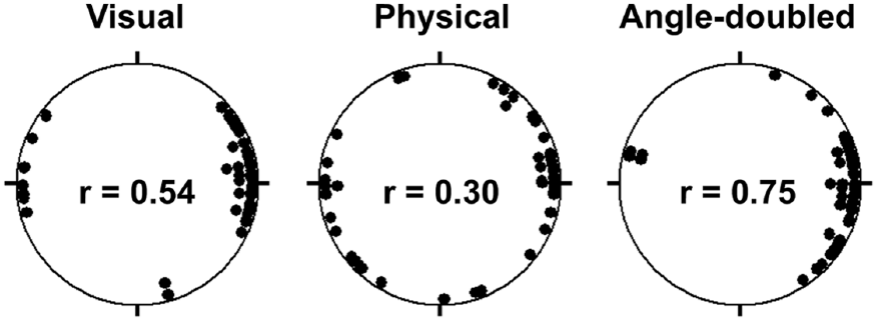
Reversal of firing provoked by visual change alone. Data from a rat in which cues were changed in visual identity independently of their physical identity, by rotating the cue cards so that a vertical bar became horizontal or a top bar became bottom, etc. When activity was aligned relative to the visual cue, activity was clustered, but slightly bipolar. When aligned to the physical cue identity, firing was more dispersed. When angles were doubled to remove bipolarity (which has the same final effect for both reference frames), the data were highly clustered around zero, indicating overall good control by the cue pair. See text for session statistics.

For five recording sessions (48 trials) from one rat (the sham LGN-lesioned one), a test was made to see whether the discrimination was purely based on visual pattern, or whether there might have been a contribution from the olfactory/tactile components of the cue cards. This was done using the V-H and T-B cue cards, which could be reversed in visual identity (V to H, or T to B, and vice versa) by rotating them around their centre points (90° to make V become H, and 180° to make T become B). Data were then analysed with firing aligned in the visual-cue reference frame (where Cue 1 was the V or T cue, irrespective of physical card identity) or in the physical reference frame (where Cue 1 is the same card across trials, regardless of its orientation). The alignment of PFDs is shown in Figure 7. In both reference frames there was a degree of bipolarity, indicating that the PFD sometimes followed the secondary cue in a given reference frame, but cue control was stronger in the visual reference frame (R-vector of 0.54) than in the physical one (R = 0.30). When R-vectors were compared across sessions, there was a decline in the score when the visual-frame data were compared with the physical-frame data (visual = 0.54 ± 0.05; physical = 0.31 ± 0.08). This difference only just reached statistical significance (t(4) = 2.2, one-tailed p = 0.046), but the improvement from single to double-angle R-scores hints at a contribution from both sensory modalities, with visual being slightly stronger.

## RSC and PoS HD cells produced unipolar tuning curves even when the landmarks were identical

No evidence of bipolar tuning curves was seen by visual inspection. However, to check quantitatively, an autocorrelation was run on the data (see section ‘Methods’) in order to determine whether there was a secondary peak at 180° due to periodic reversal of the tuning curve arising from cue confusion. Session data were combined into a single dataset for a given cell, and the autocorrelation peaks extracted. Identical-cue trials were analysed separately because there were too few of them: a two-way ANOVA of the remaining conditions found no significant differences, either as a function of cue type (F(4, 48 = 0.26, p = 0.90), autocorrelation peak (F(1, 12) = 0.59, p = 0.46) or interaction (F(4, 48 = 0.11, p = 0.36). A paired t-test on the identical-cue condition, where bipolarity might have been strongest if it occurred, was also non-significant (t(12) = 1.54, p = 0.07). Thus, there was no hint of bipolarity in the tuning curves within a single trial.

## Stabilisation of PFD across a trial

**Figure 8.**
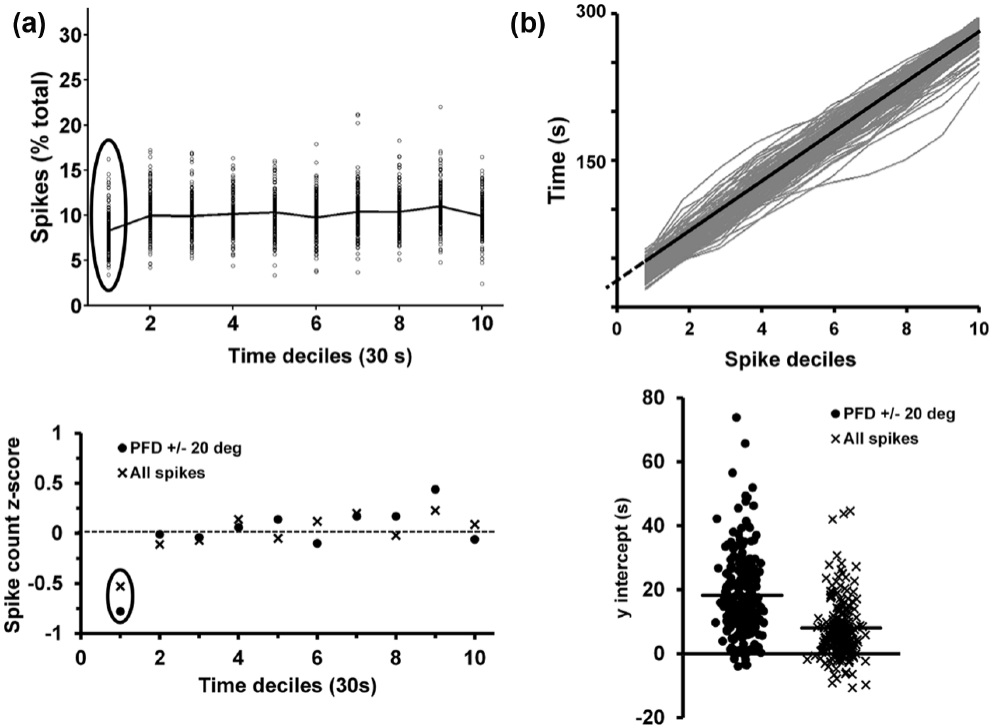
Stabilisation of the preferred firing direction (PFD) on environment entry. Data from a 40° range surrounding the PFD were divided into deciles of either time (30 s bins) or spikes (10% total). (a) Top: Spike count as a function of time decile revealed a reduced spike count in the first 30 s (circled). Bottom: z-score analysis of those data revealed a larger negative score for the first decile relative to the others, present for both spikes in the PFD range and to a lesser extent in all the spikes. See Results for statistical tests. (b) Top: The spikes from each 300 s trial were divided into 10 deciles, and the time to reach each decile plotted. Each fine grey line represents the plot from one cell over one session (averaged across trials); the solid black line is the mean across all cells/sessions and the dotted line extrapolates to the y-axis. Bottom: the y-intercepts, which should be at zero, were clustered around a mean (black line) of 18 s for the within-PFD spikes, indicating that it took extra time to reach the first decile relative to subsequent deciles. As with the spike count data, the effect was attenuated but still present when all spikes were considered.

The time course of the establishment of firing direction was computed using a stabilisation analysis (see section ‘Methods’), looking at either percentage of total spikes emitted in each 30-s time decile, or time taken to achieve each spike count decile (Figure 8). The percentage of spikes emitted in the first 30 s was 8.27 ± 0.13%, which was significantly less than the expected 10% (t(230) = 13.11, p < 0.0001). The z-score for this decile was −0.78, while for the remaining deciles it did not deviate more than 0.44 from zero (Figure 8(a)). A corresponding observation emerged from the y-intercept analysis. The plots in Figure 8(b) are highly linear, with R values close to 1 for most sessions (0.9979 ± 0.0002 for the 231 cells × sessions overall). However, the regression lines did not go through zero: the y-intercepts averaged 18.22 +/- 1.05 s, which was significantly different from zero (t(230) = 21.44, p < 0.0001). Thus, the time taken to accumulate the first decile’s worth of spikes in the PFD range was almost 20 s longer than would be expected based on the subsequent deciles. In order to determine whether this effect was due to dispersion of firing around multiple directions or just to a general decline in spiking, we repeated the analysis using all spikes, not just the ones in the PFD range; a similar, albeit slightly attenuated effect was evident (Figure 8), suggesting a general reduction of activity, rather than spatial dispersion, in the first 20–30 s of environment exploration. We looked at movement correlates including distribution of HD with respect to the PFD, linear velocity and AHV, and did not find any difference between the first decile and subsequent ones that could explain this observation (not shown). This slight reduction in spiking suggests that on entry into an environment there is a period of time during which the system is acquiring the information needed to drive the cells to firing threshold.

We compared these measures between PoS and RSC but found no differences. The total percentage of spikes accumulated in the first 30 s of the trial did not differ between PoS and RSC (t(229) = 1.86, p = 0.06) and the y-intercept measure was also not different (t(229) = 1.87, p = 0.06). We finally looked at the two measures as a function of cue type. The spike count percentages in the first 30 s did not differ between HIGH, MOD and LOW cues (F(2, 228) = 0.2, p = 0.83). The y-intercept measure showed a barely significant difference (F(2, 228) = 3,1, p = 0.048), but this went in the opposite direction from expected (highly discriminable cues had a higher y-intercept, at 21.14 s) and may have been due to the low n (n = 17) for the LOW cues.

In summary, the stabilisation analysis revealed a slight tendency for a slower accumulation of spikes (in other words, slower development of firing), especially in the PFD, in the first 20–30 s of each trial, but this did not depend on brain region or cue type. Overall, this analysis reveals that actually there was a propensity for firing to become established very soon after entry into the environment, even for cues that were harder to discriminate.

## Discussion

This study compared the activity of PoS and RSC HD cells in a landmark discrimination setting, in which two opposing landmarks had similar overall shape and/or contrast/luminance but differed in their fine-grained visual structure. The study was motivated by previous findings that lesions to the PoS or RSC impair both directional tuning stability and landmark control of HD cells in other parts of the circuit, suggesting a role for these structures in directional landmark processing. The aims were twofold: (a) to see whether the two regions differed in their responsiveness, which might provide clues to their function, and (b) to see whether HD cells could distinguish the landmarks by maintaining a consistent directional relationship to the array, and if so, to determine how quickly this discrimination occurred, and whether there were regional differences. These two considerations are discussed in turn.

### Firing properties of PoS versus RSC HD neurons

The basic firing properties we saw were similar to those reported previously, with the exception that the peak firing rates we observed in PoS were considerably lower than those that have been reported previously: ~8 Hz in our study compared with ~35 Hz reported by Taube et al. (1990b) and Taube and Muller (1998) and 19 Hz for Sharp (1996). The reason for the large discrepancies between studies might be due to the recording methods used, and the possible difficulty of the single-wire recording methods of earlier studies to identify low-firing-rate neurons. In support of this, a more recent study using tetrodes, as in the present study, found a rate of 3 Hz in PoS HD cells (Brandon et al., 2012). We also averaged across several trials, which may have brought the rates towards the mean.

Relatively few differences in firing properties were seen in HD cells from the two brain regions, but those that we observed replicated previous observations: (a) firing rates were higher in RSC; (b) tuning curves were slightly broader and (c) RSC firing anticipated HD by about 48 ms. The observation of higher firing rates in RSC is consistent with a report by Sharp (2005), who found similar but smaller differences in firing rate between these regions. We also found that firing rate was weakly (albeit significantly) correlated with running speed, with no differences between PoS and RSC. However, PoS was slightly more influenced by AHV although modulation was low for both regions.

The observation that RSC neurons anticipate upcoming HD’s, in our case by 48 ms, is similar to that published previously. Anticipatory firing was first reported by Blair and Sharp (1995) for anterior thalamic HD neurons, which were found to predict future HD by around 37 ms; this contrasted with PoS which did not show anticipation. Taube and Muller (1998) reported a value of around 23 ms for anterior thalamus, and −7 ms (firing lagging HD) for PoS HD neurons. In a later study, RSC HD neurons were also found to show anticipation, of around 25 ms (Cho and Sharp, 2001). In this study, we also saw no anticipation in PoS neurons. The implication is that RSC firing very slightly precedes PoS firing; it may be, therefore, that the route of movement-related information flow is from RSC to PoS. This is consistent with the observation that granular RSC neurons project to layer III PoS (Kononenko and Witter, 2012), which is where many HD cells in this region are found (Boccara et al., 2010; Preston-Ferrer et al., 2016), the remainder being in the deep layers (Boccara et al., 2010; Preston-Ferrer et al., 2016; Ranck, 1984; Taube et al., 1990b). Anticipation may reflect vestibular or motor efference copy signals, providing advance warning of upcoming head directions which can then be combined with descending landmark-related sensory inputs. A study of ADN neurons found that anticipatory firing also occurred, and indeed more prominently, in association with passive head movements (Bassett et al., 2005), suggesting a stronger vestibular contribution (Van der Meer et al., 2007). Clark et al. (2010) found that neurotoxic RSC lesions increase anticipatory firing in the ADN, possibly due to removal of an environment-anchoring stimulus and consequent overweighting of the vestibular AHV inputs. The precise contributions of the various self-motion signals, including how they may be differentially weighted in different species and/or under different conditions, remains to be determined (Cullen, 2014).

Because of the connections of both regions with the hippocampal formation, and because some theoretical models of HD cell processing require spatial inputs (Bicanski and Burgess, 2016), we looked for spatial correlates of firing in the form of place fields, or at least of reliable spatial heterogeneity. Both brain regions yielded some cells showing weak spatial encoding, but in general spatial modulation was low. For PoS, this observation is consistent with previous studies from this area in which HD cells typically have neither place nor theta-frequency modulation (Sharp, 1996). However, Cacucci et al. (2004) and Boccara et al. (2010) did report conjunctive encoding in regions of presubiculum very close to PoS – it is thus likely that there are regional differences in the occurrence of the difference cell types. Overall, however, it seems unlikely that spatial inputs provide a strong input to the HD cell signal directly (although the possibility exists that spatial inputs gate landmark inputs and thus exert their influence in a more subtle manner).

### Landmark discrimination

One purpose of this study was to examine possible differences in landmark processing by the two regions. Cells from both regions in all the different cue conditions had unipolar tuning curves that were clustered around a single direction relative to the cue pair, indicating that they could distinguish the cues from the background and reliably orient by them. This was true even for the visually identical cues; there was no hint that within a trial, the cells flipped their firing from one direction to its polar opposite. There are two possible reasons for this – one is that the rats could still distinguish the cues by non-visual means (e.g. perhaps by olfaction) and did in fact fire in a unipolar fashion with respect to the cues. This seems unlikely since there was confusion between cues even for the ones that were moderately discriminable; also, for the one rat in which the cues were identified by the experimenter and visual and physical identities switched between trials, the firing directions were less well predicted by physical identity. The other, more likely reason is that firing was stabilised within a trial by self-motion signals. In other words, having initially guessed at one of the two possible orientations of the cue pair on entry into the environment, the cells were then anchored to that orientation with the help of self-motion cues (since the rat knew, from these cues, that it had not suddenly reversed direction). Henceforth, the system could use the ambiguous landmark pair together with the self-motion cues to generate a stable directional signal, whereby the landmarks would prevent the signal drifting, and the self-motion cues would prevent it from flipping. This proposition is consistent with the operation of attractor dynamics, as proposed both by attractor network models (Knierim and Zhang, 2012) and following population recordings of HD cells (Peyrache et al., 2015; Seelig and Jayaraman, 2015).

Having established cue control by the landmark pair, we then looked to see whether the cues within the pair could be further discriminated. We found that discrimination of the non-identical cues was highest for the high-contrast HIGH (black/white) cue pair but was also well above control levels for the patterned, moderately discriminable (MOD) cues too. For one rat, we dissociated visual and olfactory characteristics of the cue cards and found a clear contribution from vision. These data show that these cortical HD regions, which are one synapse away from the visual cortex (Vogt and Miller, 1983; Van Groen and Wyss, 1992), can access information about internal structure of landmarks – furthermore, discrimination occurs almost immediately upon entry into the environment, and remains stable thereafter.

When we looked at the period of time immediately after environment entry we found a gradual increase in firing rate for the first few seconds, reflected in a longer time to accumulate the first decile’s worth of spikes relative to subsequent deciles. Thus, it seems that landmarks do not purely cause directional clustering of a randomly distributed base level of activity, but add drive to the system over and above baseline. Two previous studies have looked at the dynamics of cue control and have found that although it takes a significant amount of time – minutes – for a novel cue to gain control of HD cells (Goodridge et al., 1998), cells respond to rotation of a familiar cue within a few tens of milliseconds (Zugaro et al., 2003). Our situation is different because although the landmarks were generally familiar, measurements were made within the first few seconds of entry into the environment, and also the cues needed to be discriminated. This could be due to the slow onset of intrinsic network stabilisation, or it may be that a number of cognitive factors come into play that influence HD cell firing in these first few seconds – the animal may need time to adjust to the situation and start attending to spatial cues, there may be a lag while the cues are identified and the discrimination is made and so on. It would be interesting to look at firing rate if the animal were placed into the arena without the cues, to try and tease apart these possibilities.

The rapidity with which landmark discrimination was established, in the absence of explicit training, suggests that this two-cue HD cell paradigm may be a good experimental method with which to test visual discrimination capabilities of rats: for example, following lesions to visual brain areas. Because explicit training may bias animals to focus on single features of multidimensional stimuli (Jeffery, 2007), spontaneous recognition is preferable, but such processes may be hard to detect in behavioural output. Cell activity on the other hand is directly observable and easily measured. The fact that HD cells rapidly detect and discriminate complex stimuli means that they may serve as a useful read-out for sensory perception occurring within seconds or even faster.

Overall, therefore, we found relatively few differences between PoS and RSC in their cue responsiveness. The similarity in responding can be explained either by similar computations being performed on the incoming visual signals by the two regions or else by rapid communication between regions. It seems a priori unlikely that the regions perform the same computations, but to address this question in more detail it will be necessary to isolate the regions, either by lesioning each in turn or by targeted opto- or chemogenetic interventions that functionally disconnect them. This study additionally introduces a new spontaneous visual discrimination method for probing visual processing by the spatial system and reveals a basic capacity for the two cortical HD cell areas to rapidly detect and discriminate landmarks. These experiments open the door to investigations of transformation of the visual pathway following lesions or selective inactivation of inputs to these areas. Our prediction is that even when the object processing regions of the brain, such as perirhinal cortex, are removed, HD neurons will still be able, using basic visual inputs from primary visual areas, to discriminate similar landmarks and use them for orientation.

## Supplementary Material

Supplementary material
