# Supplementary material for: Retrosplenial and postsubicular head direction cells compared during visual landmark discrimination
Source: Brain Neurosci Adv. 2017 Sep 15;1:2398212817721859. doi: 10.1177/2398212817721859 (PMC6124005; doi:10.1177/2398212817721859)
Supplement: Supplementary material [file Supplementary_Material.pdf]

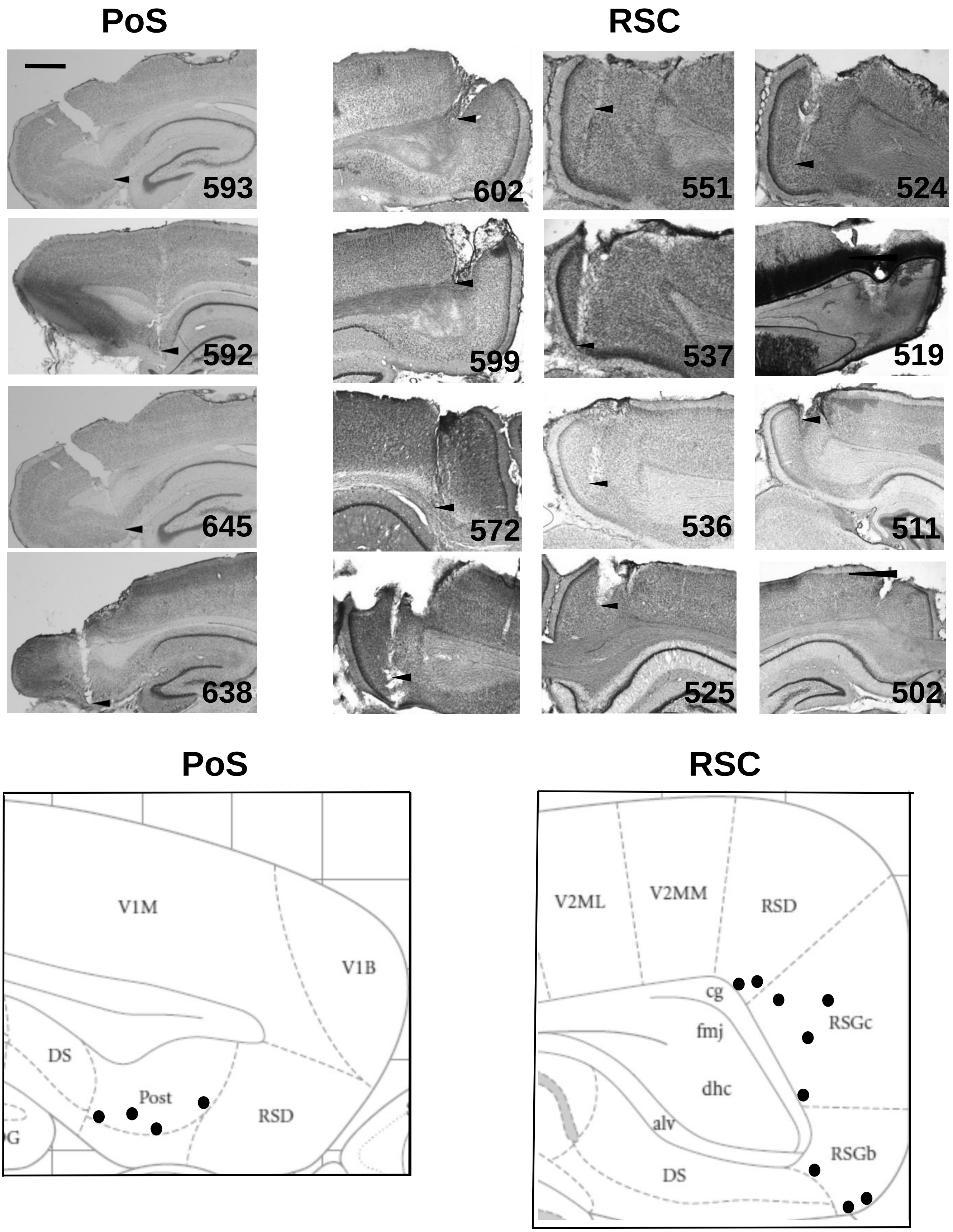

Supplementary Figure 1 Top: Coronal and sagittal sections showing the tetrode tracks of rats implanted in the PoS (first column, n = 4 due to loss of one brain) and RSC (columns 2-4; n = 12). The black triangle depicts the final electrode depth in each structure. The images were taken at 2.5x magnification with a 1 mm scale bar (black line on top left image). Bottom: Estimated recording locations superimposed on the relevant diagrams of the two areas from Paxinos and Watson (2007).

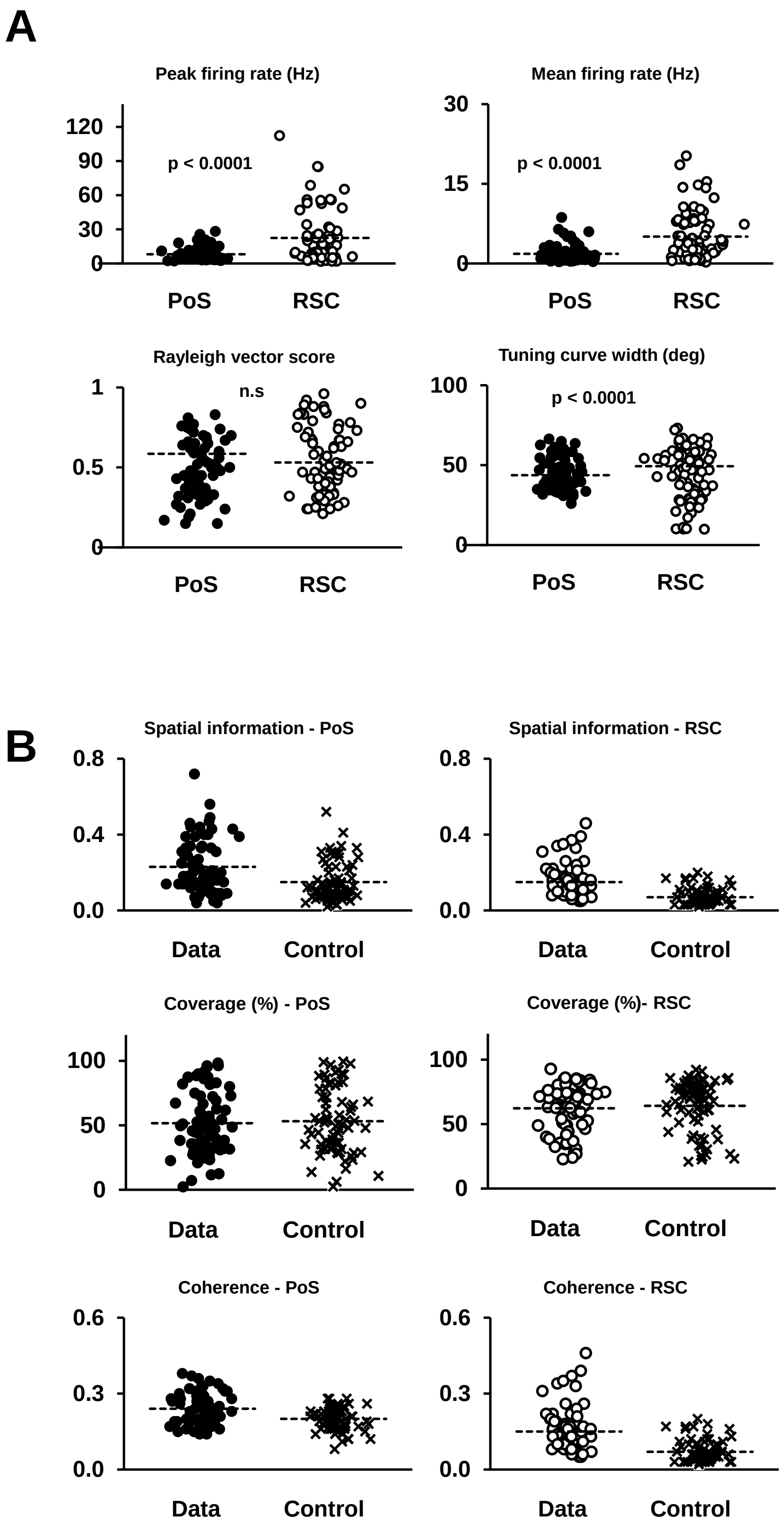

Supplementary Figure 2 (A) Basic firing characteristics of PoS and RSC HD cells. An outlier high-firing-rate cell from RSC (peak rate = 149 Hz) has been removed for graphical clarity but was included in the analyses. Dotted lines show mean values; n.s. = non-significant p-value. (B) HD cells from both PoS and RSC showed higher spatial information and greater coherence of firing and less coverage.

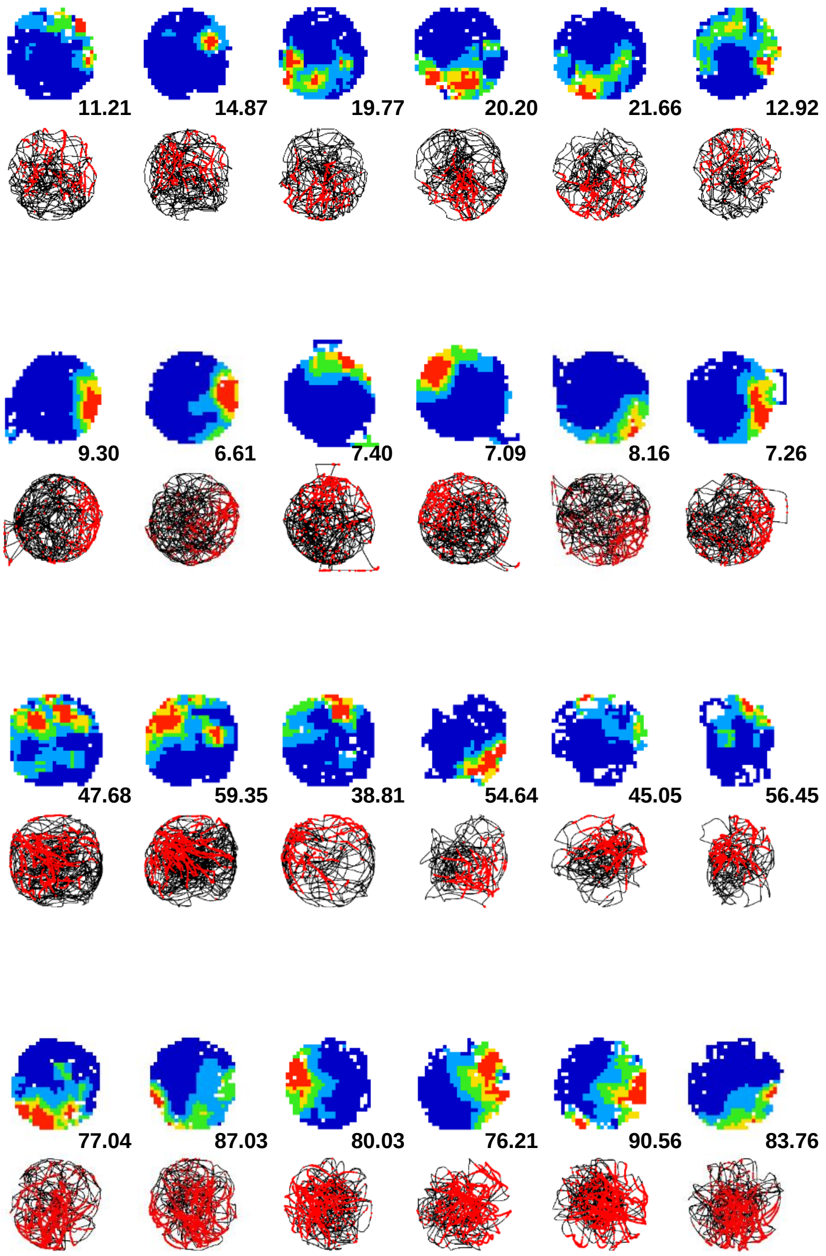

Supplementary Figure 3 Four examples of spatially modulated head direction cells in 6 trials, from PoS (top two) and RSC (bottom two). The two rows are, respectively, a firing rate map of the spatial distribution of firing (hot colours = high, cool colours = low) and a spike plot of the firing (black line = rat's path, red squares = spikes). Examples such as these were rare.

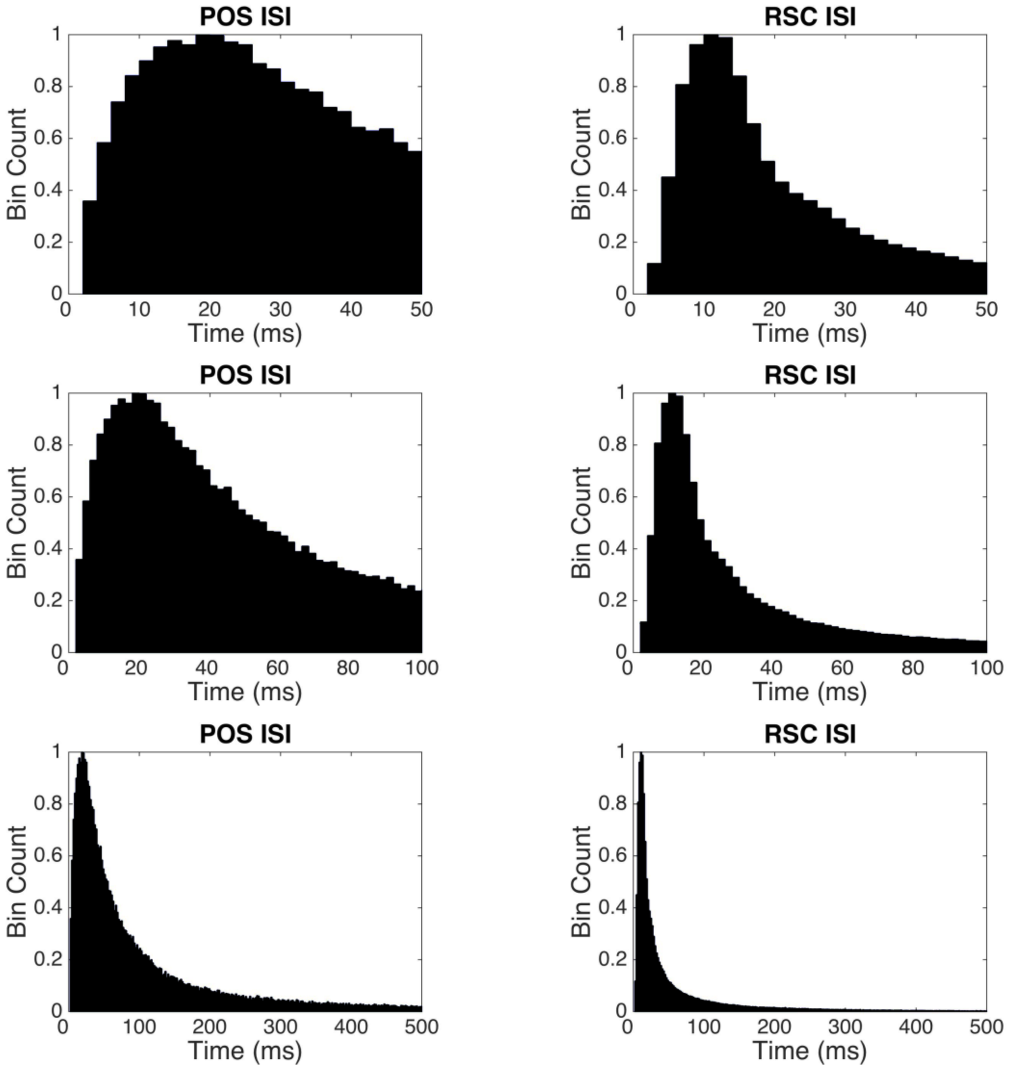

Supplementary Figure 4 Distributions of inter-spike intervals (ISIs) for the two cell types, at three different temporal resolutions. RSC neurons had an earlier peak and faster decline, reflecting their higher firing rate. No obvious periodicity (secondary peaks in the ISI) was evident for either cell type.

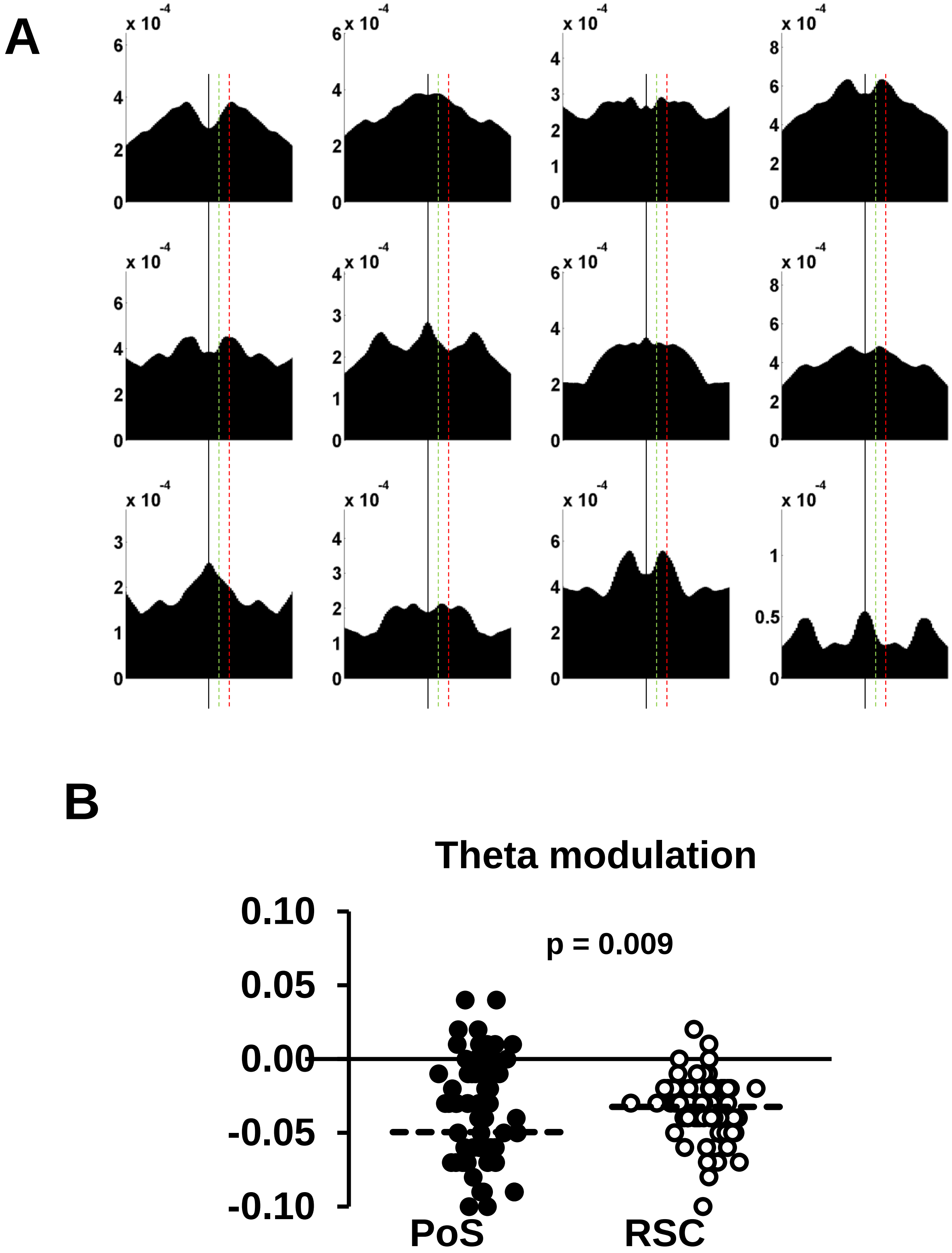

Supplementary Figure 5 Autocorrelogram analysis of theta modulation and spiking patterns. (A) Autocorrelograms to +/- 500 ms measured for a PoS HD cell across 12 trials. The theta modulation index for each cell measures the difference in height between the expected peak (red dotted line) and expected trough (green dotted line). Note that even for this one cell, periodicity and theta modulation were highly variable. (B) Cells from neither brain region showed a significant theta modulation (values above zero); the values for PoS were slightly lower and more dispersed.
